# Antibiotic administration after previable preterm prelabor rupture of membranes is associated with prolonged latency

**DOI:** 10.1002/pmf2.70342

**Published:** 2026-06-23

**Authors:** Alexandra L. Hammerquist, Alexander M. Saucedo, Selina L. Bowler, Mohan Pammi, Catherine Eppes, Ignatia Van den Veyver, Michael D. Jochum, Enrico R. Barrozo

**Affiliations:** ^1^ Department of Obstetrics and Gynecology Baylor College of Medicine and Texas Children's Hospital Houston Texas USA; ^2^ Department of Pediatrics Baylor College of Medicine and Texas Children's Hospital Houston Texas USA

**Keywords:** expectant management, interval to delivery, periviability, PPROM

## Abstract

**Introduction:**

While antibiotics have been shown to increase the interval to delivery between rupture of membranes and delivery (latency) and improve neonatal outcomes after viable preterm prelabor rupture of membranes (PPROM), this has not been well investigated in the previable PPROM population. We aimed to investigate the association between antenatal antibiotics and latency following previable PPROM. Secondarily, we examined various maternal and neonatal outcomes. We hypothesized that the administration of antibiotics would prolong latency in pregnancies with previable PPROM.

**Methods:**

Single‐center retrospective cohort study that included pregnancies diagnosed with previable PPROM between 14^0/7^ and 21^6/7^ and delivered between 2012 and 2024. Administration of antibiotics at any time after membrane rupture to prolong latency was the exposure of interest. Chi‐squared tests were performed for categorical variables and the Wilcoxon rank‐sum test for numerical variables. Statistical significance was defined as *p <* 0.05. Multivariable linear regression was performed for the primary outcome, including gestational age at rupture of membranes as a covariate.

**Results:**

After controlling for estimated gestational age at rupture of membranes, receipt of antibiotics after previable PPROM was associated with a 14.7‐day increase in latency (SE = 6.86, *p = *0.034). Antibiotics were associated with a later gestational age at delivery (25.07 vs. 20.31 weeks’ gestation) and delivery at or beyond 22^0/7^ (80.95% vs. 15.62%). Administration of antibiotics was associated with lower rates of intrauterine fetal demise (IUFD; 20.63% vs. 65.62%, *p <* 0.001) and higher rates of neonatal survival to discharge (49.21% vs. 6.25%, *p <* 0.031).

**Conclusion:**

Receipt of latency antibiotics after previable PPROM was associated with prolonged latency in both singleton and multiple gestation pregnancies. Those exposed to antibiotics had more deliveries at or beyond 22 0/7 weeks, higher neonatal survival‐to‐discharge rates, and fewer IUFDs. These findings highlight the potential benefit of latency antibiotics in extending pregnancy duration and improving neonatal outcomes following previable PPROM, supporting their consideration in expectant management and the necessity for larger prospective studies.

## 1 INTRODUCTION

Preterm prelabor rupture of membranes (PPROM) is defined as rupture of fetal membranes before onset of labor prior to 37 0/7 weeks’ gestation and complicates 2%–3% of pregnancies. Previable PPROM, defined as prelabor rupture of membranes prior to viability, is rarer, occurring in less than 1% of all pregnancies [1]. The term “previable” designates the gestational age at which survival after delivery is not possible despite intensive neonatal care. However, the gestational age of viability is not easily defined and varies between institutions. Viability in the setting of previable PPROM is primarily driven by gestational age at delivery, but also depends upon medical advancements, availability of perinatal intensive care resources, and fetal weight [1, 2]. In most institutions in the United States, 24 0/7 weeks is considered the gestational age of postnatal viability, but in some institutions where appropriate resources are available, a trial of resuscitation may be offered as early as 22 0/7 weeks per patient preference after counseling [3]. Even in advanced care settings, previable PPROM is associated with significant fetal and neonatal morbidity and mortality, including fetal or neonatal demise, necrotizing enterocolitis (NEC), intraventricular hemorrhage (IVH), respiratory distress syndrome, and pulmonary hypoplasia. Maternal risk is also significant, with complications including intraamniotic infection, endometritis, placental abruption, hemorrhage, retained placenta, and in some cases, maternal sepsis, ICU admission, or death [4, 5, 6, 7]. Furthermore, management of previable PPROM for those patients who meet criteria for expectant management is not standardized.

The goals of expectant management for PPROM after viability include prolonging the interval between rupture of membranes and delivery, referred to as latency, optimizing fetal intact survival, while also protecting maternal health. The administration of broad‐spectrum antibiotics (latency antibiotics) in expectant management of preterm PROM at 24 0/7 weeks and beyond has become the standard of care because it prolongs and reduces neonatal morbidity [6, 8]. The Society for Maternal‐Fetal Medicine (SMFM) and American College of Obstetricians & Gynecologists (ACOG) note that data on latency antibiotics before 24 0/7 weeks are scarce, limiting the ability to offer clear guidance about their administration. While they suggest that antibiotics can be considered after 20 0/7 weeks, they provide no recommendation prior to this gestational age [3]. To date, there are no published randomized trials specifically addressing the role of antibiotic therapy in previable PPROM.

The available studies on pregnancy and neonatal outcomes in the previable PPROM population report conflicting results. A 2000 study reported an association between neonatal survival and prenatal exposure to antibiotics [9], but a subsequent study found no such association [10]. Two recent studies described longer latency after antibiotic exposure, and one reported more viable deliveries [11, 12].

Given the limited data and the potentially significant effect of antibiotics in previable PPROM, the objective of this study was to investigate the association between antenatal antibiotics and latency. Secondarily, we sought to evaluate various maternal and neonatal outcomes. We hypothesized that administration of antibiotics would be associated with prolonged latency in pregnancies with previable PPROM.

## 2 MATERIALS AND METHODS

This is a single‐center, retrospective cohort study of all pregnancies diagnosed with previable PPROM between 14 0/7 and 21 6/7 weeks’ gestation who delivered at a tertiary academic institution between January 1, 2012, and December 31, 2024. Patients were identified by querying the hospital's database for appropriate Internaltional Classification of Diseases, ninth revision (ICD‐9) and International Classification of Diseases, tenth addition (ICD‐10) codes. Patients were included if diagnosed with rupture of membranes by a physician or advanced care practitioner. Diagnosis was determined at the discretion of the provider and included techniques such as visualization of pooling of amniotic fluid on speculum examination, use of cervicovaginal secretion immunoassays, visualization of ferning on microscopy, and/or nitrazine test strips. Gestational age was determined by ACOG's best obstetric criteria [13]. Multifetal gestations were also included. Exclusion criteria included patients who chose termination at any point in pregnancy; were receiving antibiotics for an indication other than PPROM; had a contraindication to expectant management at diagnosis or within 48 h of diagnosis including signs or symptoms of intraamniotic infection, abruption, preterm labor, or advanced cervical dilation; or had incomplete delivery or neonatal data, major fetal anomalies requiring significant postnatal intervention, life‐limiting anomalies, or significant pregnancy complications such as twin–twin transfusion syndrome. This protocol was approved by Baylor College of Medicine Institutional Review Board (IRB# H‐56287). We utilized the Strengthening the Reporting of Observational studies in Epidemiology (STROBE) checklist for reporting purposes.

Demographic data, delivery information, and maternal and neonatal outcomes were abstracted from patient charts. At our institution, previable PPROM management typically consists of diagnosis and evaluation in the obstetric triage department or inpatient setting. If patients are eligible and elect for expectant management, they are discharged to home, counseled on return precautions, instructed to perform daily temperature readings, and followed in outpatient clinic weekly. In this setting, neonatal resuscitation is discussed as the patient reaches the 22^nd^ week. After estimated fetal weight assessment and consultation with neonatology, if desired, patients are admitted as early as 21 weeks and 5 days for antenatal corticosteroids. Patients may elect to continue outpatient management if stable. Before January 2023, a trial of resuscitation was offered as early as 22 0/7 weeks on a case‐by‐case basis; however, after this point it became the Neonatology department's standard practice to offer intervention to all patients according to their preference after counseling. All neonates born at 24 0/7 weeks and beyond received resuscitation per institutional protocol.

The exposure of interest was the administration of antibiotics at any time after membrane rupture for the purpose of prolonging latency. The antibiotic regimen of choice was one dose of 1 g oral Azithromycin, 2 g IV Ampicillin every 6 h for 48 h, followed by 250 mg oral Amoxicillin every 8 h for 5 days. There were some deviations from this regimen due to patient allergies, latency less than 7 days, or prescription of only intravenous or only oral antibiotics. Patients were considered recipients of latency antibiotics if they received at least 2 days of antibiotics that were intended to prolong latency as the sole purpose as documented in a history and physical or progress note by the provider. Comparison groups were made between those patients who received latency antibiotics and those who did not.

The primary outcome was latency after membrane rupture. Fetal and neonatal secondary outcomes included delivery at a gestational age greater than or equal to 22 0/7 weeks, intrauterine fetal demise (IUFD), neonatal demise, neonatal death, and neonatal survival to discharge. Among live‐born neonates, the incidence of IVH, bronchopulmonary dysplasia (BPD), NEC, retinopathy of prematurity, periventricular leukomalacia, early onset sepsis, culture‐proven sepsis, spontaneous intraperitoneal perforation, Neonatal Intensive Care Unit (NICU) length of stay, early neonatal mortality, and neonatal mortality were investigated.

Maternal secondary outcomes consisted of maternal morbidity, including cesarean delivery, incidence of abruption, need for postpartum curettage, postpartum hemorrhage, maternal sepsis, intraamniotic infection, and endometritis.

Statistical analyses were performed within R version 4.4.1 in RStudio (2024‐06‐14).12.1. Chi‐squared tests were performed for categorical variables, and the Wilcoxon rank‐sum test for numerical variables. Statistical significance was defined as *p <* 0.05. Multivariable linear regression was performed for the primary outcome, including estimated gestational age at rupture of membranes (EGA at ROM) as a covariate.

## 3 RESULTS

**FIGURE 1:**
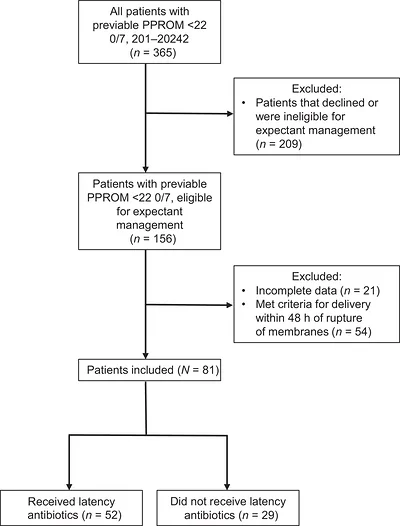
Study population. From 2012 to 2024, 365 patients had previable PPROM at less than 22 0/7 weeks’ gestation. Of these, 209 (57%) either declined expectant management or did not meet criteria to proceed with expectant management. Of the remaining 165 (43%), 54 (33%) developed an indication for delivery within 48 hour of rupture of membranes and were excluded. Patients with incomplete delivery or neonatal information (*n* = 21, 6%) were also excluded. Of the remaining 81 (51%) patients, 52 (64%) received latency antibiotics and 29 (36%) did not. PPROM, preterm prelabor rupture of membranes.

Electronic medical record query identified 365 patients with previable PPROM between 14 0/7 and 21 6/7 weeks. Of these, 209 (57%) had a contraindication to expectant management or elected for termination. Of the remaining 165 (43%), 54 (33%) spontaneously delivered or developed a contraindication to expectant management within 48 hour of rupture of membranes and were excluded. We excluded 21 patients (13%) with incomplete information about the delivery or neonatal outcomes. This left 81 patients (51%) who met the inclusion criteria, of which 18 (22%) had multiple gestation pregnancies. Patients who experienced delayed interval twin pregnancies were included. We compared data from 52/81 (64%) patients who received latency antibiotics to data from 29/81 (36%) patients who did not receive latency antibiotics (Figure 1). Maternal demographics and baseline characteristics are detailed in Table 1 and were similar between groups.

**TABLE 1:** Maternal demographics.

	No antibiotics *n *= 29 (%)	Antibiotics *n *= 52 (%)	χ‐squared	W‐statistic	*p* value
Age (years)	33 ± 6	32 ± 6		2249.75	0.246
Ethnicity/Race					
Asian	1 (3.44)	2 (3.85)	<0.001		>0.999
Hispanic Black	0 (0.00%)	2 (3.85)	0.104		0.750
Hispanic/Race Not Reported	8 (27.59%)	11 (21.15)	0.146		0.700
Hispanic White	1 (3.45%)	0 (0.00)	0.100		0.770
Multiracial/Other	0 (0.00%)	2 (3.85)	0.104		0.750
Non‐Hispanic White	2 (6.90%)	10 (19.23)	1.370		0.240
Non‐Hispanic Black	17 (58.62%)	25 (48.08)	0.460		0.500
Payor status					
Government	6 (20.9)	10 (19.23)	<0.001		>0.999
Private	23 (79.31)	42 (80.77)			
Hypertensive disease	25 (86.21)	40 (76.92)	0.510		0.470
Diabetic disease	24 (82.75)	48 (92.31)	0.890		0.346
Multifetal gestation	6 (20.7)	13 (25.0)	0.027		0.869

*Note*: Summary of relevant maternal demographics and clinical considerations. Continuous variables are presented as mean ± standard deviations (SD), with comparisons assessed with Wilcoxon test. Categorical variables are displayed as counts (*n*) and percentages (%), with comparisons evaluated using the chi‐square test. Statistically significant differences are denoted in bold (*p *< 0.05).

**FIGURE 2:**
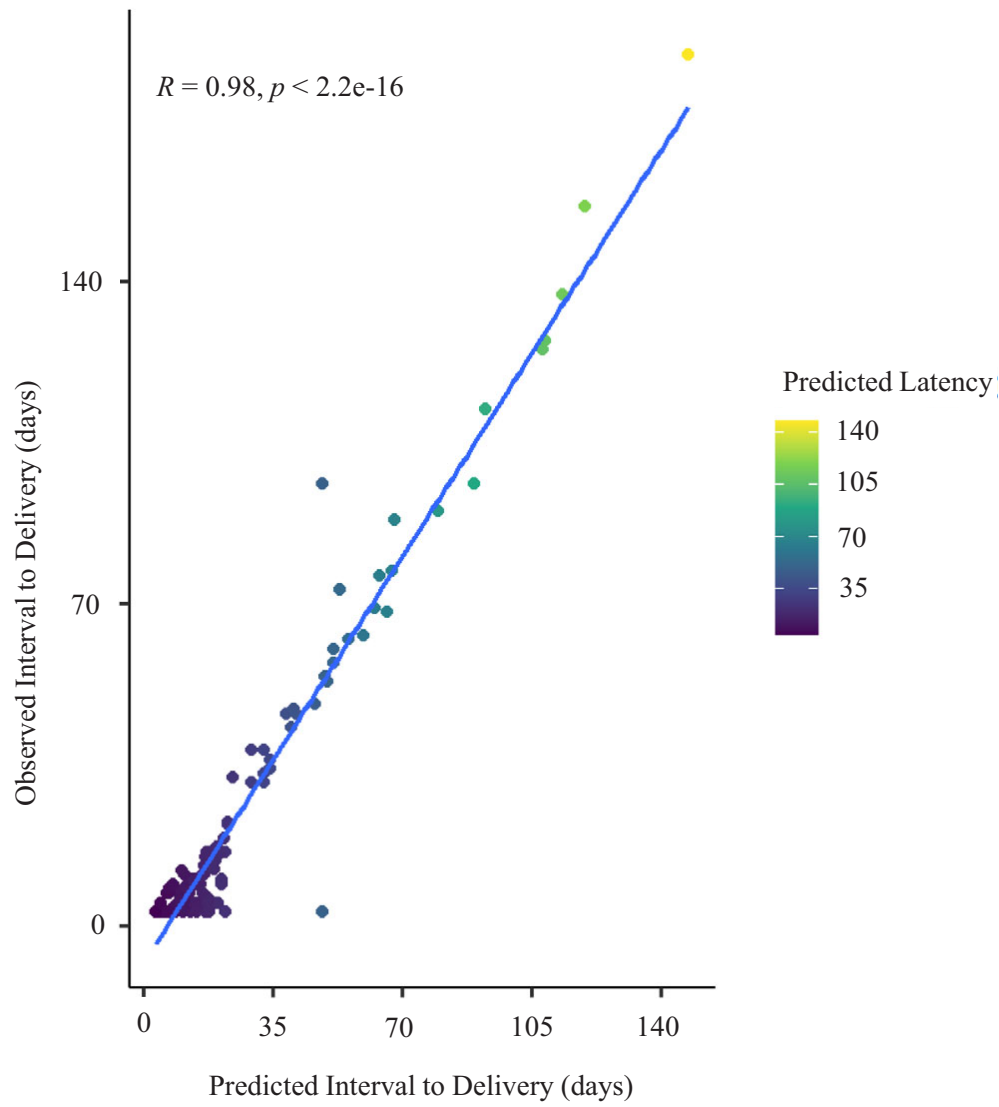
Latency antibiotics after previable PPROM are associated with prolonged latency and higher likelihood of birth after 22 weeks. Quantile–quantile plot showing strong correlation between predicted and observed latency (*r* = 0.98, *p* < 0.05) in all pregnancies, including multiples, from the multivariable model. PPROM, preterm prelabor rupture of membranes.

The primary outcome and relevant neonatal outcomes are shown in Table 2. A multivariable linear regression including EGA at ROM as a covariate (*β* = ‐0.94, SE = 0.225, *p *< 0.001) revealed that the interval from ROM to delivery was significantly longer by 14.7 days (SE = 6.86, *p = *0.034) for patients who received latency antibiotics compared to those who did not (Figure 2).

**TABLE 2:** Primary outcome and pertinent fetal and neonatal outcomes.

	No antibiotics (mean ± SD)	Antibiotics (mean ± SD)	χ‐squared	W‐statistic	*p* value
Gestational age at ROM	18.64 ± 1.97	19.62 ± 2.12		3326.5	**0.017**
Latency (days)	11.62 ± 20.23	38.15 ± 33.87		396.000	**<0.001**
Gestational age at delivery	20.31 ± 3.0	25.07 ± 3.7		253.500	**<0.001**

	No antibiotics *n *= 32 (%)	Antibiotics *n *= 63 (%)			
Delivery ≥22 weeks	5 (15.62%)	51 (80.95%)	34.773		**<0.001**
Male fetal sex	19 (59.4%)	35 (55.6%)	0.019		0.890
Intrauterine fetal demise	21 (65.62%)	13 (20.63%)	16.785		**<0.001**
Neonatal survival to discharge	2 (6.25%)	31 (49.21%)	4.674		**0.031**
Neonatal death	10 (31.25%)	15 (23.81%)	0.060		0.802

*Note*: Primary outcome and pertinent neonatal outcomes among all pregnancies, including those pregnancies that experienced fetal demise. Continuous variables are presented as mean ± standard deviations (SD), with comparisons assessed with Wilcoxon test. Categorical variables are displayed as counts (*n*) and percentages (%), with comparisons evaluated using the chi‐square test. Statistically significant differences are denoted in bold (*p *< 0.05).

Abbreviation: ROM, rupture of membranes.

We investigated the timing of antibiotic administration among those who received latency antibiotics. Latency was longer in those who received latency antibiotics later in pregnancy (*R* = 0.75, *p *< 0.05, Figure S1). The average latency after administration of antibiotics was 24.37 ± 22 (range 0.01–94.03) days. The median was 15.03 (IQR 6.02‐41.02) days.

**FIGURE 3:**
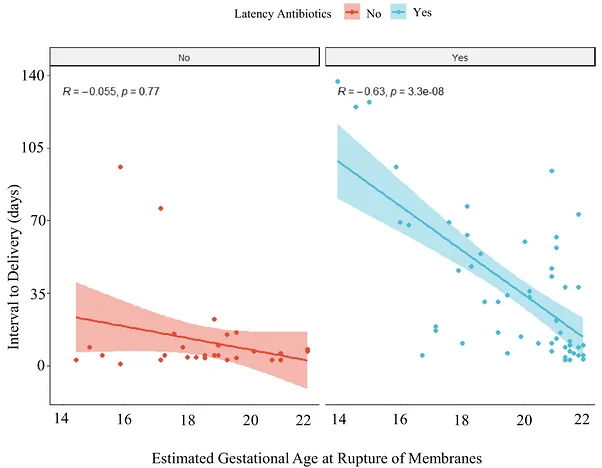
Latency shortens with advancing gestational age at rupture and is prolonged by latency antibiotics. Scatter plots show pregnancies that received latency antibiotics (blue) versus pregnancies that did not (red). Each group shows a strong negative correlation between gestational age at rupture and latency. The antibiotic group has a steeper slope, indicating greater latency earlier in gestation. Differences narrow as rupture approaches viability. Wilcoxon test, *p <* 0.05. All pregnancies are included, including multiple gestations.

Stratification by EGA at ROM revealed that latency shortens with advancing EGA at ROM, regardless of antibiotic administration. However, patients who received antibiotics had a more extreme Pearson's correlation coefficient (Figure 3; *ρ* = −0.63, *p <* 0.001) compared to those who did not (Figure 3; *ρ* = −0.06, *p = *0.770). The difference in latency between those who received antibiotics and those who did not was greater when PPROM occurred at an earlier gestational age. As gestational age approached 22 weeks, the observed latency difference between the two groups decreased.

**FIGURE 4:**
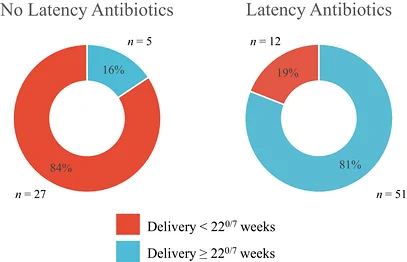
Viable delivery was associated with administration of latency antibiotics. Ring charts illustrating the difference in the number of deliveries at 22 0/7 weeks and beyond between pregnancies that received latency antibiotics (right) versus pregnancies that did not (left). Deliveries at 22 0/7 weeks or beyond are designated in blue, and deliveries at less than 22 0/7 weeks are designated in red. Chi‐square test, *p <* 0.05. All pregnancies are included, including multiple gestations.

Patients who received antibiotics delivered on average at a later gestational age (25.07 vs. 20.31 weeks) and delivered more often at or after 22 0/7 weeks (Figure 4; 80.95% vs. 15.62%; *p *< 0.05). This group had lower rates of fetal demise (20.63% vs. 65.62%, *p <* 0.001) and higher rates of neonatal survival to discharge (49.21% vs. 6.25%, *p <* 0.031) (Table 2). All neonates in this population were admitted to the NICU and developed respiratory distress syndrome. Among those live‐born neonates, there were no differences in NICU length of stay, BPD, IVH, NEC, periventricular leukomalacia, spontaneous intraperitoneal perforation, sepsis, retinopathy of prematurity, early neonatal mortality, or neonatal mortality (Table 3).

**TABLE 3:** Live‐born neonatal outcomes.

	No antibiotics *n *= 6 (%)	Antibiotics *n* = 51 (%)	χ‐squared	W‐statistic	*p* value
BPD	2 (33.33%)	20 (39.22%)	<0.001		1
IVH	1 (16.67%)	21 (41.18%)	0.523		0.470
Periventricular leukomalacia	0 (0.00%)	4 (7.84%)	<0.01		1
NEC	0 (0.00%)	6 (20.63%)	0.698		0.403
Retinopathy of prematurity	2 (33.33%)	28 (54.90%)	0.323		0.570
Early onset sepsis	0 (0.00%)	1 (1.96%)	<0.01		1
Culture proven sepsis	1 (16.67%)	8 (15.69%)	<0.001		1
SIP	1 (16.67%)	5 (9.80%)			
Early neonatal mortality (First 7 DOL)	4 (66.67%)	14 (27.45%)	0.750		0.387
Neonatal mortality (First 28 DOL)	4 (66.67%)	16 (31.37%)	1.419		0.234
LOS (72 h–90 days)	1 (16.67%)	7 (13.73%)	<0.001		1

*Note*: Neonatal outcomes among live‐born neonates. All live‐born infants were admitted to the NICU and had some degree of respiratory distress. Continuous variables are presented as mean ± standard deviations (SD), with comparisons assessed with Wilcoxon test. Categorical variables are displayed as counts (*n*) and percentages (%), with comparisons evaluated using the chi‐square test. Statistically significant differences are denoted in bold (*p *< 0.05).

Abbreviations: BPD, bronchopulmonary dysplasia; DOL, day of life; IVH, intraventricular hemorrhage; LOS, length of stay; NEC, necrotizing enterocolitis; ROM, rupture of membranes; SIP, spontaneous intraperitoneal perforation.

Regarding maternal outcomes, the latency antibiotics group had more cesarean deliveries. There were no differences between groups in placental abruption, need for postpartum curettage, incidence of postpartum hemorrhage, average estimated blood loss (EBL) at delivery, incidence of maternal sepsis, or need for product transfusion (Table 4). There was no difference in the rate of clinically diagnosed intraamniotic infection or endometritis (34% vs. 42%, *p = *0.650). There were no maternal deaths in either group.

**TABLE 4:** Maternal outcomes.

	No antibiotics (mean ± SD)	Antibiotics (mean ± SD)	χ‐squared	W‐statistic	*p* value
Average EBL at delivery	770.48 ± 986.36	746.125 ± 556.97		524.000	0.380

	No antibiotics *n *= 29 (%)	Antibiotics *n *= 52 (%)			
Cesarean delivery	8 (27.59%)	35 (67.31%)	7.19		**<0.05**
Abruption	6 (21.43%)	14 (26.42%)	0.050		0.822
Clinical and/or histopathologic intrauterine infection (*n* = 65)	24 (82.76%)	41 (78.84%)	0.017		0.894
Clinical intrauterine infection (*n* = 32)	10 (34.48%)	22 (42.31%)	0.206		0.650
Postpartum curettage	8 (27.59%)	6 (11.32%)	2.448		0.118
Maternal sepsis	1 (3.44%)	1 (1.89%)	<0.001		1.000
Postpartum hemorrhage	6 (20.69%)	11 (20.75%)	<0.001		1.000
Required transfusion	3 (10.71%)	3 (5.66%)	0.144		0.704

*Note*: Summary of relevant maternal and pregnancy outcomes. Continuous variables are presented as mean ± standard deviations (SD), with comparisons assessed with Wilcoxon test. Categorical variables are displayed as counts (*n*) and percentages (%), with comparisons evaluated using the chi‐square test. Statistically significant differences are denoted in bold (*p *< 0.05).

Abbreviation: EBL, estimated blood loss.

## 4 DISCUSSION

We found that receipt of latency antibiotics was associated with increased latency of approximately 15 days after previable PPROM in patients stable for 48 h after ROM between 14 0/7 weeks and 22 0/7 weeks. Whether this prolongation is clinically significant depends on gestational age at rupture and the likelihood of reaching viability. Accordingly, we evaluated delivery at greater than or equal to 22 0/7 weeks and found that antibiotic administration was associated with delivery beyond viability. Increased cesarean delivery rates in the antibiotic group likely reflect more patients reaching viability and pursuing neonatal resuscitation. Patients exposed to antibiotics also had fewer IUFDs and more neonates who survived to discharge. While we evaluated neonatal morbidity among live‐born neonates and no differences were identified, this analysis was limited by a small sample size.

Treatment with broad‐spectrum antibiotics after PPROM beyond 24 weeks has been shown to prolong pregnancy and reduce newborn morbidity [14, 15]. However, previable PPROM is rare, limiting prospective investigation and resulting in unclear guidance regarding antibiotic administration in this population [1, 3].

A retrospective cohort study published by Xiao et al. evaluating PPROM before 25 weeks found that antibiotic administration was more common among pregnancies with neonatal survivors (100% [*n* = 16] vs. 75% [*n* = 9]; *p = *0.05), although the antibiotic regimen was not specified [9]. Conversely, Grisaru‐Granovsky et al. found no association between ampicillin and erythromycin and neonatal survival, though the sample size was limited by a small sample size (8 vs. 14, *p = *0.527) [10]. These studies are difficult to compare given the differing inclusion criteria and small cohorts.

Dotters‐Katz et al. demonstrated that ampicillin and azithromycin initiated within 48 h of previable PPROM were associated with increased latency after controlling for gestational age at rupture (*N* = 77, *p = *0.033, HR = 0.57, 0.33, 0.97). Additionally, they reported later EGA at delivery among those exposed to antibiotics [11]. Similarly, Lambert et al. found that IV azithromycin and ampicillin followed by oral amoxicillin prolonged latency by 1.8 weeks (2.4 [1.3, 4.4.] weeks) and increased EGA at delivery [12]. Our observations are consistent with these observations.

One notable difference between our study and prior studies is the inclusion of both singleton and multiple gestations. EPIPAGE‐2 found no difference in latency between singleton and twin gestations after rupture between 22 and 25 weeks [16]. In contrast, a 2025 study reported shorter latency and worse neonatal outcomes in twin gestations after PPROM [17]. Another study evaluating latency in twin gestations with previable PPROM found no difference in latency or neonatal outcomes between treated and untreated pregnancies (0.8 vs. 2.4 weeks, *p = *0.210) [18]. While we found no difference in latency between singleton and multiple gestations, we did find that latency was longer in cases of earlier previable PPROM than in those of later previable PPROM and that antibiotics appeared to make a greater impact on latency at earlier gestational ages. This contrasts with EPIPAGE‐2, but is consistent with other previous studies [15, 16, 19].

As current guidelines remain unclear regarding administration of latency antibiotics after previable PPROM, the optimal timing of antibiotic administration of antibiotics is also uncertain. Prior studies primarily investigated the impact of antibiotics if given within 48 h of rupture [11, 12]. One study specifically comparing antibiotic initiation within 24 h versus after 24 h found no difference between these groups [20]. In our cohort, later antibiotic administration was associated with longer latency. However, this likely reflects confounding by indication, as only those who remained pregnant for longer received antibiotics later in pregnancy. To further characterize this relationship, we calculated latency after antibiotic administration and found a mean of 24.37 days and a median of 15.02 days, though wide variability limits the precision of these results.

Our finding of fewer IUFDs with latency antibiotics has not been demonstrated in prior studies. Increased neonatal survival to discharge was also observed by Dotters‐Katz et al. [11]. Similar to prior studies, we did not identify significant maternal benefit or harm associated with latency antibiotics for previable PPROM, though this analysis may be limited by sample size [11, 12, 18]. EBL was high in both groups. While there was no significant difference between groups, these values may reflect the high‐risk nature of this diagnosis.

Prospective investigation is necessary but may be challenging given the rarity of this diagnosis. Development of a regional or national previable PPROM database may help facilitate future studies. Additional future directions include placental histopathology investigation to better elucidate mechanisms underlying the duration of latency, including potential inflammatory or infectious pathways.

Our study has several strengths. This is one of the few studies evaluating latency antibiotics and previable PPROM, and to our knowledge, it includes the largest cohort to date. Inclusion of both singleton and multifetal gestations improves generalizability, and physician chart review enhances data accuracy and interpretation.

The major limitations of our study are potential confounding by indication and selection bias. Due to the retrospective design, we could not reliably determine why some patients received antibiotics or the rationale for timing of administration. Patients who received antibiotics had a later EGA at ROM, likely reflecting clinician selection of patients perceived to have a more favorable prognosis. Therefore, antibiotic administration may be a marker of patient stability, rather than a direct cause of prolonged latency. Selection bias is also possible, as patients who delivered within 48 h or were not candidates for expectant management were excluded, potentially inflating neonatal survival rates.

Additional limitations include our definition of viability as 22 0/7 weeks, which is not generalizable to lower‐resource settings. Despite representing the largest cohort currently available, the study may be underpowered for secondary outcomes. Furthermore, morbidity among patients delivering within 48 h or with contraindications to expectant management was not evaluated. Lastly, antibiotic regimens were not standardized and remained at clinical discretion, consistent with prior studies [9, 11, 12].

## 5 CONCLUSION

After controlling for gestational age at ROM, this retrospective study found a significant association between antibiotic administration and latency in a cohort of previable PPROM cases. Importantly, this cohort included both singleton and multiple gestations and included only those patients who did not spontaneously deliver or develop a contraindication to expectant management within the first 48 h after ROM. In our cohort, patients who received latency antibiotics were more likely to deliver at a later gestational age and deliver after 22 0/7 weeks. These patients had fewer IUFDs and higher neonatal survival to discharge rates. Together, these findings highlight the potential benefit of latency antibiotics in extending pregnancy duration and improving neonatal outcomes following previable PPROM, though our results may be affected by confounding by indication. While our observations support the consideration of latency antibiotics in expectant management of previable PPROM, our study, together with prior studies, points to a need to perform larger, prospective studies before latency antibiotics may be recommended clinically. In the interim, our findings may aid clinicians in counseling patients upon diagnosis of previable PPROM, given the limited guidance available for this diagnosis.

## AUTHOR CONTRIBUTIONS

**Michael D. Jochum**: Methodology; investigation; writing—review and editing; visualization; validation; software; formal analysis. **Mohan Pammi**: Writing—review and editing; data curation; supervision; methodology; resources. **Enrico R. Barrozo**: Supervision; writing—review and editing; funding acquisition; investigation; formal analysis; visualization. **Selina L. Bowler**: Data curation; writing—review and editing; resources. **Ignatia Van den Veyver**: Supervision; writing—review and editing; methodology. **Alexandra L. Hammerquist**: Conceptualization; investigation; writing—original draft; methodology; validation; writing—review and editing; visualization; formal analysis; data curation; resources; project administration. **Alexander M. Saucedo**: Conceptualization; methodology; writing—review and editing. **Catherine Eppes**: Supervision; writing—review and editing; methodology.

## CONFLICT OF INTEREST STATEMENT

The authors declare no conflicts of interest.

## Supporting information



Supporting Information

Supporting Information
