# Supplementary material for: Antibiotic administration after previable preterm prelabor rupture of membranes is associated with prolonged latency
Source: Pregnancy (Hoboken). 2026 Jun 23;2(4):e70342. doi: 10.1002/pmf2.70342 (PMC13344306; doi:10.1002/pmf2.70342)
Supplement: Supplementary file 1 [file PMF2-2-e70342-s001.docx]

**Supplemental Fig 1. Administration of latency antibiotics later is pregnancy was associated with prolonged latency.** Scatter plot with 95% confidence intervals demonstrating the observed correlation between timing of antibiotic administration after previable PPROM and total latency in days. The strong positive correlation (*R*=0.75) demonstrates that those who received antibiotics later in pregnancy had longer latency. Pearson’s r, *p*<0.05.
